# Multisystem inflammatory syndrome in neonates (MIS-N): an updated systematic review

**DOI:** 10.3389/fped.2024.1382133

**Published:** 2024-07-04

**Authors:** Divya Muthiah, Ming Chan, Yue Wey Low, Sheena Nishanti Ramasamy, Zubair Amin, Pauline Poh Lin Chan-Ng, Jeen Liang Low, Jia Ming Low

**Affiliations:** ^1^ Ministry of Health Holdings, Singapore, Singapore; ^2^ Department of Nursing, Changi General Hospital, Singapore, Singapore; ^3^ Department of Paediatrics, Khoo Teck Puat—National University Children’s Medical Institute, National University Hospital, National University Health System, Singapore, Singapore; ^4^ Department of Neonatology, Khoo Teck Puat—National University Children’s Medical Institute, National University Hospital, National University Health System, Singapore, Singapore; ^5^ Hwa Chong Institution, Singapore, Singapore

**Keywords:** multisystem inflammatory syndrome, COVID-19, neonates, pregnancy, cardiorespiratory dysfunction

## Abstract

**Introduction:**

The aim of the study was to summarize and update clinical features and outcomes of multisystem inflammatory syndrome in neonates (MIS-N).

**Methods:**

A systematic literature search was conducted of studies on MIS-N published in PubMed, MEDLINE, EMBASE, CNKI, and WHO COVID-19 databases between 1 December 2019 and 30 June 2023. Reference lists of selected articles, Google Scholar, and pre-print servers were searched for additional studies. The methodological quality of included studies was assessed.

**Results:**

Of 1,572 records screened after the initial search, 35 studies involving a total of 201 neonates with MIS-N were included. One study was retrieved from a pre-print server. For those with available data, 34/47 (78.7%) mothers were infected in the third trimester. Of the 199 mothers (two with twin pregnancies), 183 (92.0%) were from India. The median age of neonates at presentation was 2.0 days (interquartile range 1.0–9.5). Over two-thirds (144/201, 71.6%) presented with respiratory distress, while 112 (55.7%) had cardiac involvement, such as ventricular dysfunctions, involvement of coronary arteries, and atrioventricular blocks. Arrhythmias and thrombosis were reported in 15/201 (7.5%) and 2/201 (3.0%) neonates, respectively. All neonates, except one, required critical care; 64/160 (40.0%) required inotropic support and 105/187 (56.1%) required respiratory support, of whom 59/105 (56.2%) were specified to require intubation. The mortality rate was 5.0% (10/201).

**Discussion/Conclusion:**

MIS-N should be considered in ill neonates presenting with involvement of two or more organ systems, especially among those neonates with cardiorespiratory dysfunctions, in the presence of proven or suspected maternal COVID-19 infection during pregnancy.

**Systematic Review Registration:**

https://www.crd.york.ac.uk/prospero/display_record.php?ID=CRD42021278717, PROSPERO, identifier CRD42021278717.

## Introduction

Transplacental transfer of severe acute respiratory syndrome coronavirus-2 (SARS-CoV-2) antibodies from mother to fetus has generally been thought to be protective against SARS-CoV-2 infection (1). However, this transfer of antibodies, along with *in utero* transfer of other inflammatory cytokines (or the response to antibodies mounted by the neonate to SARS-CoV-2 infection) may, in rare cases, trigger a process similar to multisystem inflammatory syndrome in children (MIS-C), with the potential to cause severe immune activation, manifesting as a multisystem inflammatory syndrome in neonates (MIS-N) (2, 3).

In the initial phase of the pandemic, there was no formal or clear definition of MIS-N, in part due to a scarcity of data as well as difficulty in diagnosis due to many overlapping symptoms (2, 4). In the absence of specific laboratory tests for MIS-N, a diagnosis was thus suspected based on clinical signs and symptoms, together with ancillary laboratory findings. Pawar et al. were the first to distinguish MIS-N as a distinct post-infectious immune-mediated syndrome in infants born to mothers with SARS-CoV-2 infection contracted during pregnancy from complications of postnatally acquired primary COVID-19 (5). Although the specific pathogenesis of MIS-N is unknown, two mechanisms have been postulated: (1) transplacental transfer of maternal antibodies; and (2) vertical transmission of maternal infection resulting in endogenous production of antibodies in the fetus (2, 3). These antibodies then initiate a cascade of exaggerated inflammatory responses causing widespread tissue damage in the neonate (3).

This updated systematic review summarizes cases of MIS-N that have been reported in the literature and describes the clinical features of MIS-N.

## Methods

### Study design

A systematic review protocol was developed in accordance with the Preferred Reporting Items for Systematic Reviews and Meta-Analyses Protocols (PRISMA-P) checklist and registered in the International Prospective Register of Systematic Reviews (PROSPERO; protocol registration number: CRD42021278717) on 14 March 2021.

### Search strategy

Articles were retrieved from PubMed/MEDLINE, EMBASE, China National Knowledge Infrastructure (CNKI), and WHO COVID-19 databases. Gray literature, through Google Scholar, pre-print servers (i.e., Research Square, medRxiv), and reference lists of identified articles, was searched for additional studies of interest. Studies published between 1 December 2019 and 30 June 2023 that reported MIS-N secondary to maternal SARS-CoV-2 exposure or infection were included. The search was restricted to articles in the English language.

The search strategy for PubMed/MEDLINE used keywords and MeSH (MEDLINE) terms; this was then adapted accordingly for the other databases. Search terms included neonate, novel coronavirus, COVID-19, 2019-nCoV, SARS-CoV-2, Coronavirus infection, multisystem inflammatory syndrome in children, multisystem inflammatory syndrome in neonates, MIS-C, MIS-N, Kawasaki, Kawasaki-like, hyperinflammation, hyperinflammatory shock, vasculitis, macrophage activation syndrome, hemophagocytic lymphohistiocytosis, pediatric multisystem inflammatory syndrome, PMIS, and toxic shock syndrome. The search was performed in conjunction with a qualified medical librarian experienced in conducting systematic reviews.

### Eligibility criteria and study selection

Two reviewers (JML and MC) independently screened titles and abstracts and assessed full-text articles for inclusion. A third reviewer (YWL) resolved any disagreements on study eligibility. Study authors were contacted for clarification if information on eligibility was unavailable or unclear.

We used the following case definition of MIS-N: an inflammatory syndrome affecting neonates (≤28 days of life) with confirmed maternal SARS-CoV-2 exposure or infection during pregnancy in the absence of an alternative diagnosis, whereby there is (1) severe illness requiring hospitalization and (2) two or more organ system involvement or presence of cardiac AV conduction abnormalities/coronary artery dilatation. These had to be accompanied by laboratory evidence of elevated inflammatory markers [C-reactive protein (CRP), procalcitonin, erythrocyte sedimentation rate, lactate dehydrogenase, D-dimer, interleukin-6 (IL-6), ferritin, fibrinogen] with positive SARS-CoV-2 immunoglobulin G (IgG) in the neonate. Maternal SARS-CoV-2 infection or exposure was defined as laboratory-confirmed COVID-19 infection using either quantitative real-time reverse transcription PCR (qRT-PCR) for SARS-CoV-2, immunoassays such as ELISA for SARS-CoV-2 specific IgG/IgM, or clinical history suggestive for SARS-CoV-2 infection. Given the inconsistency of fever in neonates with MIS-N, this was not included as a criterion, unlike in MIS-C. Neonates with postnatal SARS-CoV-2 infection before the development of multisystem inflammation were excluded.

To ensure a comprehensive and up-to-date search on this topic, we determined *a priori* that the review would include case reports, case series, cohort, case–control, and cross-sectional studies that were published in peer-reviewed journal and pre-print servers. We excluded review articles or articles written based on secondary data.

### Data extraction

Two reviewers (DM and JLL) independently extracted individual participant data, such as neonatal and maternal demographics, SARS-CoV-2 test results, clinical manifestations of MIS-N, investigations, findings, treatments, and outcomes.

### Quality assessment

The quality of included studies was assessed using the framework by Murad et al., which evaluates the methodological quality of studies based on four domains (selection, ascertainment, causality, and reporting) (6). Two reviewers (DM and JLL) completed the quality assessment independently, and a third reviewer (JML) resolved any inconsistencies (Supplementary Material Table S1).

## Results

**Figure 1:**
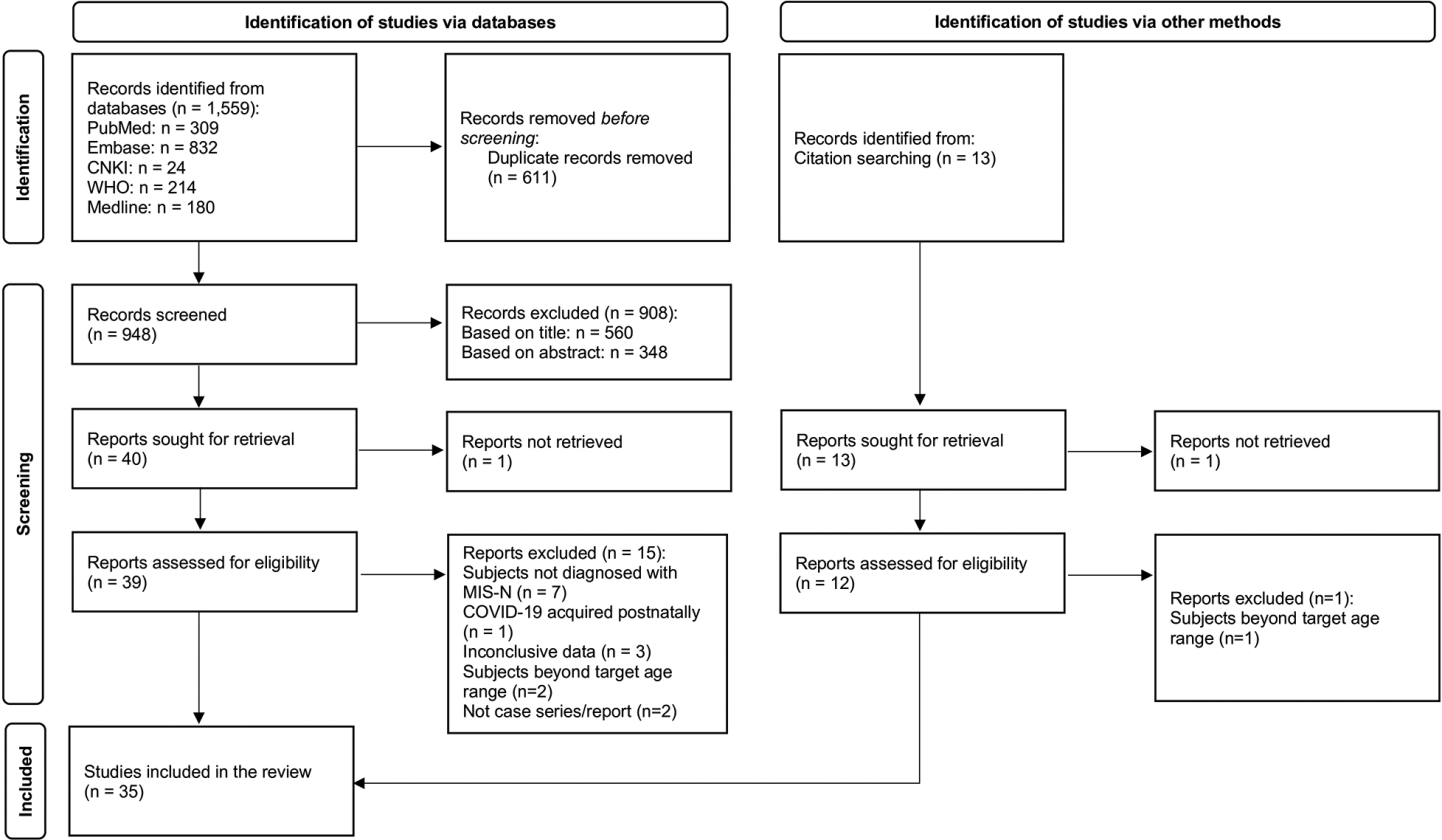
Preferred reporting items for systematic reviews and meta-analyses (PRISMA) flow diagram (40).

The search yielded 1,572 articles (records identified from databases: 1,559; records identified from citation search: 13). After removing duplicates, 961 articles were screened, with 908 articles excluded after title and abstract screening. Two reports could not be retrieved. Of the remaining 51 articles screened by full text, 35 articles, consisting of 25 case reports (7–30), 9 case series (31–38), and 1 cohort study (39) were selected (Figure 1). One study was identified from a pre-print server (39) and was added to the review in view of the rarity of cases after quality assessment. The total number of MIS-N cases included was 201; 8 additional cases were excluded as they did not meet the study criteria. There were no duplicate cases that appeared in both case reports and case series. Reported denominators in this review represent the cases for which relevant data were available.

### Quality assessment of included studies

In total, 34 studies, including the pre-print (7–22, 24–39), fulfilled all four domains of the quality assessment framework; only one study (23) fulfilled two domains. Inter-rater agreement between the two reviewers was 100%. Quality assessment for the domain of subject selection was high (100%), with a low risk of sampling bias where patients represented the whole experience of the investigator/centers. All studies confirmed maternal SARS-CoV-2 infection or exposure during pregnancy, and accurately ascertained outcome measures while excluding alternative diagnoses. All studies described cases with sufficient details for replication or allowed practitioners to make inferences related to their own practice (high quality) (Supplementary Material Table S1).

### Demographics of neonates with MIS-N and their mothers

Participant data from 201 neonates were extracted and statistically combined for analysis (Supplementary Material Table S2).

#### Demographics of neonates with MIS-N

Of the 201 neonates, 87 (43.3%) were born at term (≥37 weeks); 37/89 (41.6%) were female. The age of presentation was in the range of 1–28 days of life, with a median time of 2 days [interquartile range (IQR) 1.0–9.5]. For those with available data, 14/74 (18.9%) neonates had low birth weight (i.e., below 2.5 kg). Of 100 neonates, 46 (46%) were delivered by normal vaginal delivery and 54 (54%) by Cesarean section (Supplementary Material Table S2).

#### Demographics of mothers of neonates with MIS-N

Of the 199 mothers (two with twin pregnancies), 183 (92.0%) were from India. For those studies with reported data, over one-third (9/22, 40.9%) had comorbidities: pre-eclampsia (*n* = 3); diabetes mellitus (*n* = 4); and diabetes with pregnancy-induced hypertension (*n* = 2), of which one also had hyperthyroidism (Supplementary Material Table S2).

### Clinical presentation and course

**Figure 2:**
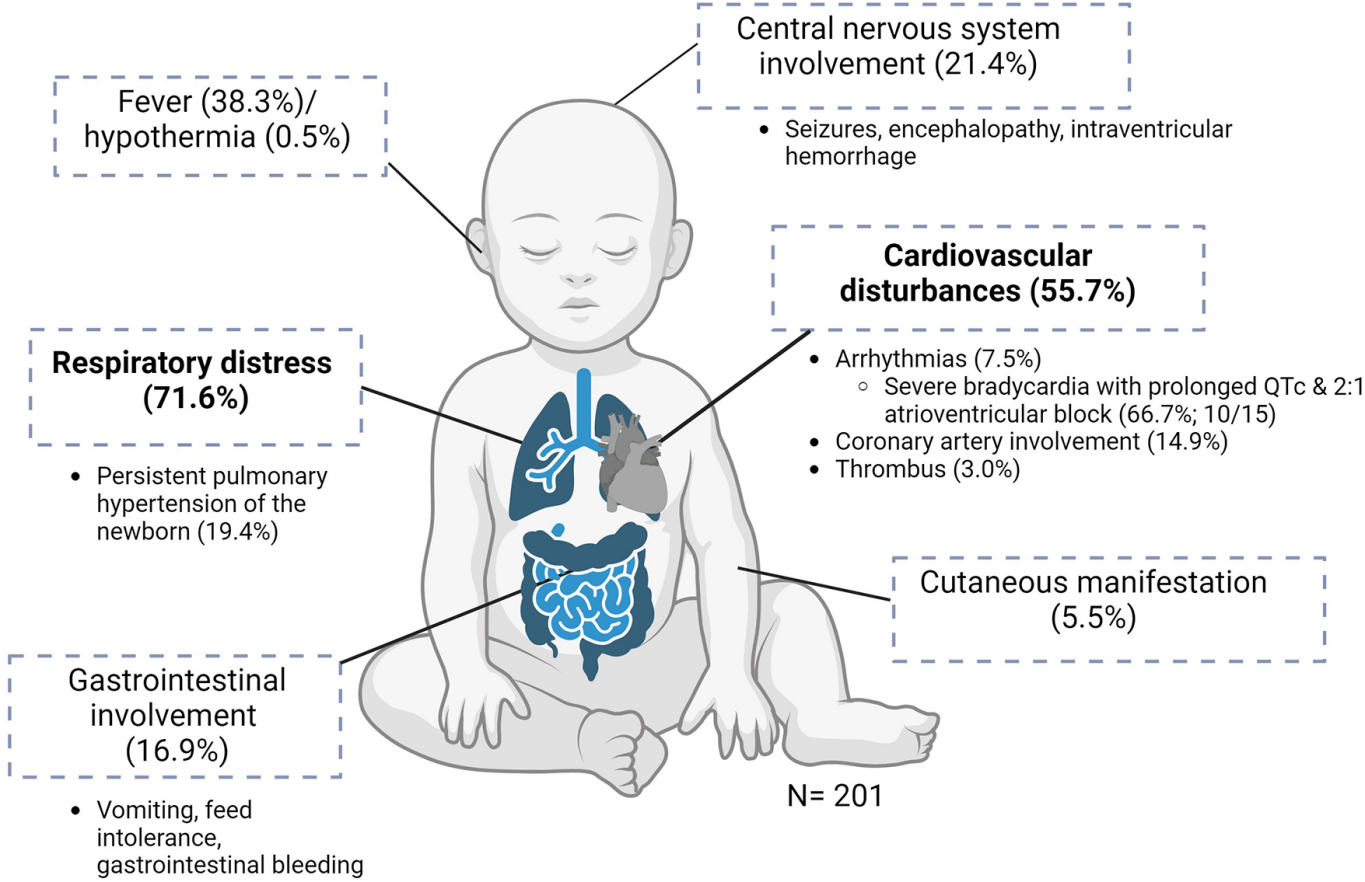
Clinical manifestations of MIS-N (*n* = 201). Created using BioRender.com.

The clinical manifestations are illustrated in Figure 2. Of the 201 neonates, 144 (71.6%) presented with respiratory distress, of whom 28 (19.4%) had persistent pulmonary hypertension of the newborn. The next most common manifestation was cardiovascular disturbances (112/201, 55.7%), including hypotensive shock. Arrhythmias were present in 15/201 (7.5%) neonates, of whom 10/15 (66.7%) had severe bradycardia with prolonged QTc interval and 2:1 atrioventricular block. Of the 201 infants, 30 (14.9%) had coronary artery involvement: 6 (3.0%) had thrombi (intracardiac (*n* = 4), aortic (*n* = 1), and pulmonary trunk (*n* = 1) (Table 1, Supplementary Material Table S3).

**Table 1:** Clinical features of MIS-N.

Study	Clinical features of MIS-N
Divekar et al. (7)	Hypoxemic respiratory failure, hypotensive shock, PPHN, pathological coronary artery dilatation, disseminated intravascular coagulation, hepatic dysfunction, renal failure with oliguria and anasarca (*n* = 1)
Lima et al. (8)	Antenatally diagnosed pericardial effusion, with postnatal hemodynamic instability, apnea, bradycardia, respiratory distress, metabolic acidosis (*n* = 1)
Kappanayil et al. (9)	Cardiogenic hypotensive shock, respiratory distress, hepatomegaly, necrotic pressure ulcers over occiput and gluteal regions (*n* = 1)
McCarty et al. (10)	Fever, PPHN, respiratory failure (*n* = 1)
Schoenmakers et al. (11)	Hypotensive shock with multi-organ failure (raised creatinine, liver and cardiac enzymes), PPHN, bilateral intraventricular hemorrhage (grade 3 left sided; grade 2 right sided), coronary artery dilatation, hepatic and renal dysfunction (*n* = 1)
Borkotoky et al. (12)	PPHN, respiratory distress, vasculitis rash, fever, necrotizing enterocolitis (*n* = 1)
Shaiba et al. (13)	PPHN, left ventricular systolic dysfunction, transaminitis (*n* = 1)
Amonkar et al. (14)	Spontaneous aortic thrombosis resulting in right lower limb gangrene, irritability (*n* = 1)
Diwakar et al. (15)	Fever, diarrhea, rash over forehead and cheeks (*n* = 1)
Costa et al. (16)	Multiple organ involvement, abnormal thickening of coronary artery walls (*n* = 1)
Amulya et al. (17)	Fever, cough, rhonchi, respiratory distress, abnormal body movements, coronary artery dilatation (*n* = 1)
Agrawal et al. (18)	Fever, lethargy, compensated shock, respiratory distress, intestinal dilatation, occipital ulcer (*n* = 1)
Bakhle et al. (19)	Fever, lethargy, respiratory distress due to cavitary lung lesion (*n* = 1)
Nitya et al. (20)	Poor activity, cold peripheries, and feeble peripheral pulses. In shock with erythematous rash over the eyelids, cheek, chest, and upper abdomen (*n* = 1)
Sojisirikul et al. (21)	Abdominal distention and apnea followed by respiratory distress, tachycardia, and tachypnea (*n* = 1)
Voddapelli et al. (22)	Fever, drowsiness, tachycardia, hypotension with cool peripheries, and tachypnea with chest retractions. Developed abdominal distension with bilious vomiting and fever spikes (*n* = 1)
Gupta et al. (23)	Refractory PPHN with persistent cardiac dysfunction and coagulopathy (*n* = 1). Unexplained severe cardiac dysfunction and hypertension on day 6 of life associated with aortic and intracardiac thrombosis (*n* = 1)
Malek et al. (24)	Respiratory distress (tachypnea, chest indrawing, grunting with peripheral cyanosis), acute kidney injury
Shinde et al. (25)	Respiratory distress, poor perfusion, capillary refill time 5 s, hypotension, tachycardia, cold extremities. Lethargic, seizures.
Aguilar-Caballero et al. (26)	Progressive respiratory deterioration
Arun et al. (27)	Fever, poor feeding, lethargy, seizures, apnea, anemia, intramuscular hematoma, intracranial bleed
Ragireddy et al. (28)	Respiratory distress, poor perfusion, poor feeding, pulmonary arterial hypertension (*n* = 1)
Rackauskaite et al. (29)	Myocarditis, supraventricular tachycardia, cardiogenic shock, hepatic injury, renal failure
Abdulaziz-Opiela et al. (30)	Respiratory distress, embolism in arterial duct, left pulmonary artery, and pulmonary trunk, pericardial effusion, ascites, hepatomegaly
Shanker et al. (31)	Fever, respiratory distress, shock, feeding difficulty, lethargy, seizure (*n* = 1)
	Feeding difficulty, lethargy (*n* = 3)
More et al. (32)	Hypotensive shock, respiratory distress, encephalopathy (*n* = 1)
	Hypotensive shock, respiratory distress, refusal of feeds, lethargy (*n* = 1)
	Hypotensive shock, respiratory distress, fever (*n* = 1)
	Apnea, refusal of feeds, lethargy, seizure (*n* = 1)
	Apnea, refusal of feeds, lethargy, fever (*n* = 1)
	Respiratory distress, refusal of feeds, lethargy (*n* = 1)
	Respiratory distress, refusal of feeds, lethargy, fever (*n* = 1)
	Refusal of feeds, lethargy, fever (*n* = 2)
	Refusal of feeds, fever (*n* = 1)
	Respiratory distress (*n* = 2)
	Respiratory distress, fever (*n* = 1)
Pawar et al. (5)	Bradycardia with prolonged QTc and 2:1 AVB (*n* = 4)
	Respiratory distress, bradycardia with prolonged QTc and 2:1 AVB (*n* = 3)
	Fever, hypotension, LV dysfunction (*n* = 1)
	Antenatal fetoplacental compromise, shock, mild LV dysfunction, bilateral pleural effusions (*n* = 1)
	Antenatal fetoplacental compromise, bradycardia with prolonged QTc and 2:1 AVB (*n* = 1)
	Antenatal fetoplacental compromise, bradycardia with prolonged QTc and 2:1 AVB, feeding intolerance (*n* = 1)
	Antenatal fetoplacental compromise. Brownish gastric aspirates, frank melena, SVT, bilateral pleural and pericardial effusion (*n* = 1)
	Grunting, tachypnea, lethargy, feeding intolerance, intermittent bradycardia, hypotension (*n* = 1)
	Feed intolerance, decreased activity, brown gastric aspirates, rash, pedal edema, oral and skin lesions (*n* = 1)
	Feed intolerance, cardiomegaly, cardiogenic shock, dilated coronaries, severe pulmonary arterial hypertension (*n* = 1)
	Seizures, shock, bradycardia, acute renal failure, mild LV dysfunction (*n* = 1)
	Fever, feeding intolerance, tachypnea, desaturation (*n* = 1)
	Antenatal pleural, pericardial effusions and ascites, respiratory distress, shock, dilated hypertrophied RV with dysfunction, large thrombus at LPA origin (*n* = 1)
	Antenatal pleural, pericardial effusions, and ascites, pitting edema over chest wall, hepatomegaly, tachypnea, crepitations, dilated coronaries (*n* = 1)
	Mottling and poor peripheral pulsations, hypotension, intracardiac thrombus in right atrium (*n* = 1)
Tambekar et al. (33)	Mild respiratory distress, tachycardia, cold peripheries, capillary refill time >3 s, grade 2 murmur, mild ascites with hepatic congestion (*n* = 1)
	Grade 2 murmur, poor feeding (*n* = 1)
	Grade 4 murmur, tachycardia, poor perfusion, poor feeding, absent suck, lethargy, seizures, hypotonia, hyporeflexia, maculopapular rash (*n* = 1)
Saeedi et al. (34)	Diarrhea, dehydration, fever, multi-form macular rash on trunk and limbs (*n* = 1)
	Cough, rashes on head/palms/soles (*n* = 1)
Balleda et al. (35)	Fever (*n* = 18)
	Respiratory distress (*n* = 17)
	Seizures (*n* = 8)
	Abdominal distension (*n* = 3)
	Skin rashes (*n* = 2)
	Coronary artery dilatation (*n* = 8)
	Renal impairment (*n* = 1)—only patient who died, involved >3 systems (respiratory, cardiovascular, neurological and renal)
Chaudhuri et al. (36)	Severe respiratory distress, shock (*n* = 1)
	Thrombocytopenia respiratory distress, coronary aneurysm, PPHN (*n* = 1)
	Respiratory distress, dilated right heart with cardiomegaly, coronary aneurysm, PPHN, ventricular dysfunction (*n* = 1)
	PPHN, shock (*n* = 1)
	PPHN, coronary aneurysm (*n* = 2)
	Intermittent premature atrial ectopics, desaturation (*n* = 1)
	Cardiogenic shock, pneumothorax, ICH, hydrocephalous (*n* = 1)
	Cardiogenic shock (*n* = 1)
	Respiratory distress, oxygen dependency, large thrombus in left atrium, coronary aneurysm, mild PAH (*n* = 1)
	Cyanosis, severe PAH, moderate TR, right-to-left shunt across foramen ovale and bidirectional ductal shunt, cardiomegaly (*n* = 1)
	Renal impairment (*n* = 5)
Hashiq et al. (37)	Hematemesis, meconium aspiration syndrome, respiratory distress (*n* = 2)
	Hematemesis, respiratory distress (*n* = 1)
	Hematemesis, seizure (*n* = 1)
Gamez-Gonzalez et al. (38)	Respiratory distress, coronary artery dilatation, pericardial effusion (*n* = 1)
	Respiratory distress, coronary artery dilatation (*n* = 1)
	Respiratory distress, pulmonary and tricuspid regurgitation, finger desquamation (*n* = 1)
Charki et al. (39)	Respiratory distress (*n* = 56)
	Cardiac dysfunction (*n* = 46)
	PPHN (*n* = 16)
	Fever (*n* = 40)
	Seizures (*n* = 15)
	Encephalopathy (*n* = 7)
	Sepsis-like (*n* = 30)
	Hypoglycemia (*n* = 4)
	Parotitis (*n* = 4)

AVB, atrioventricular block; ICH, intracranial hemorrhage; LPA, left pulmonary artery; LV, left ventricle; MIS-N, multisystem inflammatory syndrome in neonates; PAH, pulmonary arterial hypertension; PPHN, persistent pulmonary hypertension of newborn; QTc, QT corrected for heart rate; SVT, supraventricular tachycardia; TR, tricuspid regurgitation; RV, right ventricle.

Of the 201 neonates, 34 (16.9%) had gastrointestinal involvement, mostly in the form of vomiting, feed intolerance, and gastrointestinal tract bleeding. A total of 43/201 (21.4%) infants had neurological involvement in the form of seizures, encephalopathy, or intraventricular hemorrhage. Seven infants (3.5%) had evidence of extravascular fluid leakage, such as pericardial and/or pleural effusions with ascites. A total of 12 (6.0%) infants were reported to have renal impairment. Fever was present in 77/201 (38.3%) neonates and 1 (0.5%) had hypothermia. Of the infants, 11/201 (5.5%) developed cutaneous manifestations during the illness. No musculoskeletal manifestations were reported (Table 1).

### Maternal and neonatal findings, including SARS-CoV-2

#### Maternal SARS-CoV-2

Maternal SARS-CoV-2 exposure or infection was diagnosed with qRT-PCR test in 58/199 (29.1%) mothers and by serology in 133/199 (56.8%) mothers; the remainder (28/199, 14.1%) had a diagnosis made based on history of close contact with COVID-19 cases (typically a family member) (Supplementary Material Table S2).

Among those with available data, 25/55 (40.0%) mothers were symptomatic for COVID-19 during pregnancy, with upper respiratory tract symptoms and/or malaise, while 33/55 (60.0%) were asymptomatic. Only one mother had severe illness requiring oxygen supplementation or intensive care. Out of 47 mothers, 37 (78.7%) were infected during their last trimester, 7 (14.9%) in the second trimester, and 3 (6.3%) in the first trimester (Supplementary Material Table S2).

#### Neonatal SARS-CoV-2

SARS-CoV-2 IgG was detected in 191/201 (95.0%) neonates in the first month of life. Of the 10 that were not detected, 4 had lab-proven maternal history of COVID-19 with placental evidence of infection, 5 had lab-proven maternal history of COVID-19, and 1 was deemed to be positive from suggestive contact tracing; they were culture negative (12/12, 100%) for bacteria and tested negative for other viruses (Supplementary Material Table S2).

### Laboratory, electrocardiographic, and imaging findings

We report the following data made available by individual publications, hence the denominators vary. Inflammatory markers were raised in most patients, with high levels of CRP and ferritin reported in 115/185 (62.1%) and 87/161 (54.0%) neonates, respectively. Of the 46/201 (22.8%) neonates who had CRP values available, the median CRP level was 4.25 mg/dl (IQR 1.72–7.6 mg/dl; normal range <1 mg/L). Of the 201 neonates, 50 (24.8%) had ferritin values available: the median ferritin level was 873 ng/ml (IQR 371.2–1,466). Procalcitonin was raised in 19/57 (33.3%) neonates, lactate dehydrogenase in 90/182 (49.5%) neonates, and D-dimer in 123/190 (64.7%). IL-6 was found to be raised in 16/17 (94.1%) neonates who were tested, of whom 13 had the following reported values: median 36.2 (IQR 20.1–69.2; normal range 0–7 pg/ml). Out of 200 neonates, 85 (42.5%) had elevated cardiac enzymes. Hematological abnormalities included thrombocytopenia in 41/70 (58.5%) neonates and thrombocytosis in 19/70 (27.1%) (Supplementary Material Table S3).

ECG findings were reported in 32/105 (30.5%) neonates, of which there were 6 normal sinus rhythms, 12 sinus tachycardia, 2 supraventricular tachycardia, 1 atrial bigeminy, 1 non-specific ST change, and 10 bradycardias with prolonged corrected QT interval, of which 9 had 2:1 AV blocks (Supplementary Material Table S4).

Chest X-ray findings included 10 normal radiographs, 2 with cardiomegaly, 1 with bilateral pulmonary infiltrates, and 1 with diffuse hazy granular opacities. Two patients underwent a CT scan of the thorax, which showed an inflammatory ground-glass pattern in one patient and bilateral reticulonodular opacities in the other (Supplementary Material Table S3).

### Therapeutic management

All neonates, except one, required intensive care. Of 160 neonates, 64 (40%) required inotropic support and 105/187 (56.1%) required respiratory support, of whom 59 (56.2%) were specified to require intubation. Immunomodulatory therapy [intravenous steroids and/or intravenous immunoglobulins (IVIGs)] was used in 125/201 (62.2%) neonates: 104 (51.7%) received IVIG and 107 (53.2%) received steroids. Among the 107 neonates who received steroid therapy, 72 (67.3%) received intravenous methylprednisolone or prednisolone, 22 (20.6%) received dexamethasone, and 4 (3.7%) received hydrocortisone. The type of steroids, however, was not specified for 9 (8.4%) neonates. Anticoagulants were used in 33/201 (16.4%) neonates and aspirin in 30/201 (14.9%). None received biologics or convalescent plasma (Supplementary Material Table S5).

### Outcomes

The mean length of stay of the neonates was 21.86 days (range 5–150 days). The mortality rate for MIS-N was 5.0% (10/201). No long-term outcomes were reported for the patients who survived until discharge or follow-up (191/201, 95.0%). The duration of follow-up was up to 3 months (Supplementary Material Table S5).

## Discussion/conclusion

Our review summarizes the current data available on MIS-N in neonates born to mothers who contracted COVID-19 during pregnancy and distinguishes itself from previous systematic reviews published earlier in the pandemic that summarized both *in utero* and postpartum transmission of COVID-19 viral-induced, post-infective immune dysregulation in the neonatal population. Our review also serves as an update of the existing literature (2, 4). At the time of writing, another systematic review has been published, which analyzed the clinical features and management strategies of published cases of MIS-N up to 30 September 2022 (41). As there has been an increase in reported cases, we then systematically updated the search and completed a comprehensive review with statistical analyses to provide further insights and understanding of this relatively new condition.

Initial reviews of MIS-N reported a mortality rate in the range of 8.2%–11% (42); however, our current review with almost double the number of cases reported a lower mortality rate of 5% (10/201). This difference could be related to probable reporting bias for critical neonates with MIS-N in the literature in earlier part of epidemic.

We defined MIS-N as a distinct entity from neonates who develop MIS-C after COVID-19 infections contracted after birth (2, 4). This distinction from MIS-C was needed given the differences in clinical presentations between MIS-C and MIS-N. It is critical to recognize MIS-N, as all neonates afflicted with MIS-N had a more severe clinical course with almost all requiring intensive care.

We observed certain features unique to MIS-N. Unlike postnatally acquired SARS-CoV-2 infection or MIS-C, less than half of the affected neonates with MIS-N had fever (43, 44). More than two-thirds of neonates with MIS-N typically presented with cardiorespiratory dysfunction within 2 days of birth. MIS-N–affected neonates had high levels of inflammatory cytokines, such as IL-6, and other inflammatory markers, such as CRP and ferritin. However, there are no specific biomarkers for MIS-N (3). Nonetheless, these findings should be interpreted with caution as there was no control group of infants born to women without COVID-19 during pregnancy and hence cannot be concluded to be causal. Understandably, this area of work would be challenging to study given the uptake in COVID-19 vaccination status among pregnant women and the added complexity of interpreting SARs-CoV-2 antibody levels in neonates whose mothers have been vaccinated. While we continue to evaluate whether these findings are attributable to SARS-CoV2 in pregnancy, having a low threshold for ECG and ECHO in ill neonates with perplexing diagnoses seems reasonable.

We also attempted to evaluate the vaccination status of mothers of neonates with MIS-N; however, the numbers were too small to be conclusive. While the risk of MIS-N in neonates born to vaccinated mothers has not been extensively reviewed (44), emerging evidence supports the protective effect of maternal mRNA COVID-19 vaccinations during pregnancy against SARS-CoV-2 infection and its complications, including MIS-N among infants in the first 6 months of life (45). The role of transplacental transfer of antibodies in protecting infants is also seen with other recommended antenatal vaccine-preventable diseases, such as influenza (46). Future work involving neonates afflicted with MIS-N should investigate maternal vaccination status as a potential protective mechanism against MIS-N.

From a clinical perspective, the diagnosis in newborns is based on clinical suspicion, suggestive history from the mother, and suggestive lab findings. Given the rarity of this condition, a high index of suspicion is needed. If the diagnosis is suspected, we propose that a transthoracic echocardiogram, a non-invasive and relatively easily test, should be performed and infants should be monitored closely for multisystem involvement. It is imperative that concurrent diagnoses, such as neonatal sepsis and other viral infections (e.g., enterovirus infection), should be entertained and additional specific therapy, such as empirical antibiotics, should be added.

In this review, we observed that the principles of treating MIS-C were used in the treatment of MIS-N. At present, there is insufficient evidence to conclude that immunomodulatory agents, such as early initiation of steroids and IVIG, play a beneficial role in the treatment of MIS-N. More work is required to determine the efficacy of immunomodulatory treatment. Therefore, the mainstay of therapy is supportive. It is essential that adequate cardiorespiratory support, fluid and nutrition, and thermoregulation are provided. In the absence of a proper clinical trial, it is imprudent to suggest routine use of systemic steroids or other immune modulatory therapy for all cases of MIS-N, especially because of the risk of adverse side effects from systemic steroids in neonates including possible long-term neurodevelopmental impairment.

The strength of this review is the inclusion of a larger number of cases with a detailed statistically combined data analysis. This review has some limitations. First, data on some variables of interest, such as maternal COVID-19 vaccination status, were not available for all included cases. Second, the lack of universal definition could lead to a misdiagnosis or underreporting of MIS-N cases. Third, there could be reporting bias as most cases were case reports and case series from India, which form the current predominant available literature for MIS-N. We have included one study, a prospective cohort, from pre-print servers (47); which contributed to 100 cases (out of 201). Finally, the long-term outcomes of MIS-N are unknown. Further studies with larger cohorts over an extended period of time are necessary to investigate the full spectrum of clinical features and short- and long-term outcomes.

In conclusion, healthcare professionals looking after neonates born to mothers with SARS-CoV-2 infection or significant exposure should remain cognizant of MIS-N as a possible differential diagnosis in seriously unwell babies as MIS-N appears to have distinct clinical presentation, laboratory profiles, and outcomes.
