# Supplementary material for: Multisystem inflammatory syndrome in neonates (MIS-N): an updated systematic review
Source: Front Pediatr. 2024 Jul 4;12:1382133. doi: 10.3389/fped.2024.1382133 (PMC11256206; doi:10.3389/fped.2024.1382133)
Supplement: Supplementary file 1 [file Table1.docx]

**Supplementary Table 1.** Assessment of methodological quality of included studies using the framework by Murad *et al*.^8^

| **Study** | **Domains** | | | | | | | |
| --- | --- | --- | --- | --- | --- | --- | --- | --- |
|  | **Selection** | **Ascertainment** | | **Causality** | | | | **Reporting** |
|  | 1. Does the patient(s) represent(s) the whole experience of the investigator (centre) or is the selection method unclear to the extent that other patients with similar presentation may not have been reported? | 2. Was the exposure adequately ascertained? | 3. Was the outcome adequately ascertained? | 4. Were other alternative causes that may explain the observation ruled out? | 5. Was there a challenge/ rechallenge phenomenon? | 6. Was there a dose–response effect? | 7. Was follow-up long enough for outcomes to occur? | 8. Is the case(s) described with sufficient details to allow other investigators to replicate the research or to allow practitioners make inferences related to their own practice? |
| Divekar *et al*, 2021^7^ | Y | Y | Y | Y | NA | NA | Y | Y |
| Lima *et al,* 2020^8^ | Y | Y | Y | Y | NA | NA | Y | Y |
| Kappanayil *et al*, 2021^9^ | Y | Y | Y | Y | NA | NA | Y | Y |
| McCarty *et al,* 2021^10^ | Y | Y | Y | Y | NA | NA | Y | Y |
| Schoenmakers *et al,* 2020^11^ | Y | Y | Y | N | NA | NA | Y | Y |
| Borkotoky *et al,* 2021^12^ | Y | Y | Y | N | NA | NA | Y | Y |
| Shaiba *et al,* 2021^13^ | Y | Y | Y | N | NA | NA | Y | Y |
| Amonkar *et al,* 2021^14^ | Y | Y | Y | Y | NA | NA | Y | Y |
| Diwakar *et al,* 2021^15^ | Y | Y | Y | Y | NA | NA | Y | Y |
| Costa *et al,* 2021^16^ | Y | Y | Y | Y | NA | NA | Y | Y |
| Amulya *et al,* 2021^17^ | Y | Y | Y | Y | NA | NA | Y | Y |
| Agrawal *et al,* 2021^18^ | Y | Y | Y | Y | NA | NA | Y | Y |
| Bakhle *et al,* 2022^19^ | Y | Y | Y | Y | NA | NA | Y | Y |
| Nitya *et al,* 2022^20^ | Y | Y | Y | Y | NA | NA | Y | Y |
| Sojisirikul *et al,* 2022^21^ | Y | Y | Y | Y | NA | NA | Y | Y |
| Voddapelli *et al,* 2022^22^ | Y | Y | Y | Y | NA | NA | Y | Y |
| Gupta *et al,* 2022^23^ | Y | N | N | Y | NA | NA | Y | N |
| Malek *et al*, 2022^24^ | Y | Y | Y | Y | NA | Y | Y | Y |
| Shinde *et al*, 2021^25^ | Y | Y | Y | Y | NA | Y | Y | Y |
| Aguilar-Caballero *et al*, 2023^26^ | Y | Y | Y | Y | NA | Y | Y | Y |
| Arun *et al*, 2022^27^ | Y | Y | Y | Y | NA | Y | Y | Y |
| Ragireddy *et al,* 2023^28^ | Y | Y | Y | Y | NA | Y | Y | Y |
| Rackauskaite *et al,* 2023^29^ | Y | Y | Y | Y | NA | Y | Y | Y |
| Abdulaziz- Opiela *et al,* 2023^30^ | Y | Y | Y | Y | NA | Y | Y | Y |
| Shanker *et al*, 2021^31^ | Y | Y | Y | Y | NA | Y | Y | Y |
| More *et al,* 2022^32^ | Y | Y | Y | Y | NA | NA | Y | Y |
| Pawar *et al*, 2021^33^ | Y | Y | Y | Y | NA | NA | Y | Y |
| Tambekar *et al*, 2022^34^ | Y | Y | Y | Y | NA | Y | Y | Y |
| Saeedi *et al*, 2023^35^ | Y | Y | Y | Y | NA | Y | Y | Y |
| Balleda *et al*, 2022^36^ | Y | Y | Y | Y | NA | Y | Y | Y |
| Chaudhuri *et al*, 2022^37^ | Y | Y | Y | Y | NA | Y | Y | Y |
| Hashiq *et al*, 2021^38^ | Y | Y | Y | Y | NA | Y | Y | Y |
| Gamez- Gonzalez *et al,* 2022^39^ | Y | Y | Y | Y | NA | Y | Y | Y |
| Charki *et al,* 2022^40^ | Y | Y | Y | Y | NA | NA | Y | Y |
