# Supplementary material for: Multisystem inflammatory syndrome in neonates (MIS-N): an updated systematic review
Source: Front Pediatr. 2024 Jul 4;12:1382133. doi: 10.3389/fped.2024.1382133 (PMC11256206; doi:10.3389/fped.2024.1382133)
Supplement: Supplementary file 2 [file Table2.docx]

**Supplementary Table 2.** Characteristics of mother-child dyads for infants with MIS-N.

| **Study** | **Country** | **Study type; no. of cases** | **Gestational age at birth; Day of presenting illness [Median (Range)]** | **Gestational age of maternal COVID-19 infection; Trimester [Median (Range)]** | **Sex** | **Birth weight (g)**  **[Median (Range)]** | **Maternal SARS-CoV-2 diagnosis (qRT-PCR, Serology)** | **Neonatal COVID-19 serology (IgG, IgM)** | **Maternal Age & Ethnicity if stated [Median (Range)]** | **Maternal comorbidities, COVID vaccination status** | **Maternal symptoms during COVID-19 infection** | **Mode of delivery** | **Placental histology** |
| --- | --- | --- | --- | --- | --- | --- | --- | --- | --- | --- | --- | --- | --- |
| Divekar *et al*, 2021^7^ | USA | Case report; 1 | GA 30; D1 | GA 30; Third | F | 1300 | ID NOW+^b^, IgG+ IgM- | IgG+, IgM-, ART -ve | 24, Hispanic | Severe preeclampsia, substance abuse, poor prenatal care | Asymptomatic | LSCS | NS |
| Lima *et al,* 2020^8^ | Brazil | Case report; 1 | GA 33+4; D1 | GA 29; Third | F | 2400 | IgG+ IgM+ | IgG+, IgM+, RT-PCR- | 27 | None | Flu-like | LSCS | Mild nonspecific circulatory changes, with varying sizes of chorionic villi, intervening space with foci of calcification and hemorrhage, deciduous with slight deposition of fibrin and fibrinoid necrosis in the wall vessels. |
| Kappanayil *et al*, 2021^9^ | India | Case report; 1 | Term; D22 | GA 31; Third | F | 3750 | RT-PCR+, IgG+ | IgG+, IgM-, RT-PCR- | NS | NS | Flu-like | NVD | NS |
| McCarty *et al,* 2021^10^ | USA | Case report; 1 | GA 34+6; D1 | 34+6; Third | M | NS | RT-PCR+ | RT-PCR-, no serologies | 32 | Severe preeclampsia | Flu-like | NVD | Focal chronic infarcts consistent with vascular damage from inflammation due to maternal viral infection. |
| Schoenmakers *et al,* 2020^11^ | Netherlands | Case report; 1 | Preterm; D1 | NS; Third | F | 75th centile | RT-PCR+ | Serologies -ve, RT-PCR- | NS | Obese, GDM | General malaise, myalgia and fever | LSCS | Presence of SARS-CoV-2 with generalized inflammation characterized by histiocytic intervillositis with diffuse perivillous fibrin depositions; damage to syncytiotrophoblasts. |
| Borkotoky *et al,* 2021^12^ | UK | Case report; 1 | GA 38+3; D1 | GA 35; Third | M | 4840 | RT-PCR-, IgG+, IgM- | IgG+, IgM-, RT-PCR- | 41 | GDM | Fever, cough | LSCS | NS |
| Shaiba *et al,* 2021^13^ | Saudi Arabia | Case report; 1 | GA 36; D1 | 1st time: Second 2nd time: Third | F | 3004 | RT PCR+ | IgG + | 33, Filipino | None | 1st time: asymptomatic 2nd time: mild URTI | NVD | Hypocoiled umbilical cord. Chorionic villi compatible with given GA with few intervillous hematomas and scattered areas of chorangiosis. |
| Amonkar *et al,* 2021^14^ | India | Case report; 1 | Term; D6 | NS | M | 2400 | RT-PCR-, total IgG & IgM + | Total IgG/IgM+, RT-PCR- | NS | None | Asymptomatic | NVD | NS |
| Diwakar *et al,* 2021^15^ | India | Case report; 1 | GA 39; D18 | GA 38; Third | M | 3250 | RT-PCR+ | IgG+, RT-PCR - | 36 | GDM, PIH | URTI symptoms, fever | LSCS | NS |
| Costa *et al,* 2021^16^ | Italy | Case report; 1 | NS; D1 | NS | NS | NS | NS | IgG+ | NS | NS | NS | NS | NS |
| Amulya *et al,* 2021^17^ | India | Case report; 1 | NS; D10 | NS; Second | M | NS | Serology + | IgG+, IgM+ | NS | NS | NS | NS | NS |
| Agrawal *et al,* 2021^18^ | India | Case report; 1 | GA 39; D2 | GA 35; Third | M | 3300 | RT-PCR-, IgG+ IgM- | IgG+, IgM-, RT-PCR- | 34 | None; Unvaccinated | Asymptomatic | LSCS | NS |
| Bakhle *et al,* 2022^19^ | India | Case report; 1 | GA 37; D8 | GA 29; Third | M | NS | IgG+ IgM- | IgG+, IgM-, RT-PCR- | NS | None | Mild | LSCS | NS |
| Nitya *et al,* 2022^20^ | India | Case report; 1 | Term; D2 | NS; First | F | 2750 | Serology + | IgG+ | NS | None; Unvaccinated | NS | LSCS | NS |
| Sojisirikul *et al,* 2022^21^ | Thailand | Case report; 1 | GA 33; D15 | GA 19; Second | F; 2nd MCDA twin | 1230 | IgG+, IgM-, RT-PCR - | IgG+, IgM-, RT-PCR - | 34 | NS; Unvaccinated | Asymptomatic | LSCS | NS |
| Voddapelli *et al,* 2022^22^ | India | Case report; 1 | GA 35; D3 | GA 32; Third | F | 2640 | NS | IgG+, IgM-, RT-PCR - | NS | NS; Unvaccinated | NS | LSCS | NS |
| Gupta *et al,* 2022^23^ | India | Case report; 1 | Term; D1 | NS; First and possibly third | M | NS | NS | IgG+, IgM- | NS | NS | Asymptomatic | NS | NS |
|  |  | Case report; 2 | Term; D6 | Just prior to delivery | F | NS | NS | IgG+, IgM- | NS | NS | NS | NS | NS |
| Malek *et al*, 2022^24^ | Bangladesh | Case report; 1 | GA 35; D1 | GA 27; Third | F | 1950 | RT-PCR+ | RT-PCR-, no serologies | 33 | DM, PIH, hyperthyroidism | Fever, cough, SOB | LSCS | NS |
| Shinde *et al*, 2021^25^ | India | Case report; 1 | GA 32; D1 | NA | M | 1474 | IgG +, RT-PCR - | IgG+ | 26 | NS; Unvaccinated | Asymptomatic | NVD | NS |
| Aguilar-Caballero *et al*, 2023^26^ | Brazil | Case report; 1 | GA 28+5; D1 | GA 26+6; Second | M; DCDA twin | 1200 | RT-PCR+ | IgG+, IgM+ | 23 | None | Asymptomatic | NS | Placental pathology of co-twin showed acute chorioamnionitis with areas of fibrin deposition characteristic of intra-amniotic infection of an unidentified source |
| Arun *et al*, 2022^27^ | India | Case report; 1 | GA 39; D2 | GA 32; Third | M | 2800 | RT-PCR+ | IgG+ | NS | None | NS | NVD | NS |
| Ragireddy *et al,* 2023^28^ | India | Case report; 1 | GA 38; D25 | NS | M | 2850 | RT-PCR+, IgG+ | IgG+ | 32 | NS | Cough | NVD | NS |
| Rackauskaite *et al,* 2023^29^ | Lithuania | Case report; 1 | Term; D21 | NS; Third | M | NS | RT-PCR+, IgG+ | IgG+ | NS | NS | NS | NS | NS |
| Abdulaziz- Opiela *et al,* 2023^30^ | Poland | Case report; 1 | GA 40; D1 | NS | M | 2580 | RT-PCR+, IgG+ | IgG+ | NS | NS, Unvaccinated | fever,  headache,  fatigue, cough | LSCS | NS |
| Shanker *et al*, 2021^31^ | India | Case series; 4 | GA 37; D25 GA 38; D15 GA 36; D22 GA 36; D19 | GA 36; Third GA 37; Third GA 32-36; Third GA 34: Third | NS NS M NS | NS NS 2720 NS | RT-PCR+ RT-PCR+ IgG+, RT-PCR+ RT-PCR+ | NS NS RT-PCR- NS | NS | NS | NS NS Asymptomatic NS | NS NS LSCS NS | NS |
| More *et al,* 2022^32^ | India | Case series; 14 | Term ≥37w (10) Late preterm 34–36w (3)  Preterm<33w (1); D5 (D1-30) | Third (1), NS (13) | M (10), F (4) | 2560 (1500 - 3400) | IgG+ (7), RT-PCR+ (6) | IgG+ | NS | 1 unvaccinated, rest NS | NS | NS | NS |
| Pawar *et al*, 2021^33^ | India | Case series; 20 | GA 34 (27–38); Term ≥37w (3) Late preterm 34–36w (13)  Preterm<33w (4); D2 (D1-5); | GA 28 (12 – 35); First (1), Second (2), Third (15) | Male (10), Female (10); Singleton (15) Twins (5) | 2150 (1000– 4000) | IgG+ (4), RT-PCR+ (3) | IgG+ (18), IgG < cut off (2) | 26.5 (20–34) | NS, Unvaccinated | Asymptomatic (12), Fever (6) | 7 LSCS; 13 NVD | NS |
| Tambekar *et al*, 2022^34^ | India | Case series; 3 | GA 36; D1 GA 34: D1 Term; D4 | NS NS Third | F (3) | 1600 2700 2500 | IgG+ IgG+, IgM+, RT-PCR- IgG+ | IgG+, IgM + IgG+, IgM + IgG+ | 30 25 NS | None GDM NS | Asymptomatic Asymptomatic Fever, cough, cold | LSCS LSCS NS | NS |
| Saeedi *et al*, 2023^35^ | Iran | Case series; 2 | GA 39; D17 GA 38; D20 | GA 35; Third GA 30; Third | M F | NS | IgG + IgM + NS | IgG +, RT-PCR- IgG +; IgM + | 23 NS | NS | Asymptomatic | LSCS | NS |
| Balleda *et al*, 2022^36^ | India | Case series; 18 | GA 35 (1), 36 (11), 38 (6) | NS | M (11), F (7) | NS | IgG+ (18) | IgG+ (18) | NS | None | NA | NA | NS |
| Chaudhuri *et al*, 2022^37^ | India | Case series; 12 | GA 27.5 (27 - 40); D2 (1 - 4) | NS | NA | 2450 (650-3190) | IgG+ (9) RT-PCR+ (1) | IgG+ (10), RT-PCR+ (1) | NS | NS | Asymptomatic (9), Symptomatic (3) | NVD (3), LSCS (9) | NS |
| Hashiq *et al*, 2021^38^ | India | Case series; 4 | GA 36.2 GA 35 GA 35 GA 35.4 | NS | M M F M | 1700 1500 1600 1800 | Nil (4) | IgG+ (4) | NS | NS | Nil | LSCS (4) | NS |
| Gamez- Gonzalez *et al,* 2022^39^ | Mexico | Case series; 3 | NS; D1 GA 32; D1 GA 35; D1 | NS NS GA 18, Second | F M F | 3700 1300 1900 | RT-PCR+, IgG+ | IgG+ (3) | 32 NS 25 | DM Pre-eclampsia NS | Asymptomatic Respiratory distress NS | LSCS (3) | NS |
| Charki *et al,* 2022^40^ | India | Cohort study; 98 | Preterm <37w (49) Term ≥37w (49);  D1 (n=46),  D2 (n=12),  D3-7 (n=12), D8-14 (n=4), D15-21 (n=8), D22-28 (n=16) | NS | NS | NS | RT-PCR+(n=34),  IgG (n=56) | IgG+ (n=98) | NS | NS | NS | NS | NS |
