# Supplementary material for: Multisystem inflammatory syndrome in neonates (MIS-N): an updated systematic review
Source: Front Pediatr. 2024 Jul 4;12:1382133. doi: 10.3389/fped.2024.1382133 (PMC11256206; doi:10.3389/fped.2024.1382133)
Supplement: Supplementary file 3 [file Table3.docx]

**Supplementary Table 3.** Clinical features of MIS-N.

| **Study** | **Clinical features of MIS-N** |
| --- | --- |
| Divekar *et al*, 2021^7^ | Hypoxemic respiratory failure, hypotensive shock, persistent pulmonary hypertension of newborn (PPHN), pathological coronary artery dilatation, disseminated intravascular coagulation, hepatic dysfunction, renal failure with oliguria and anasarca (n=1) |
| Lima *et al,* 2020^8^ | Antenatally diagnosed pericardial effusion, with postnatal haemodynamic instability, apnea, bradycardia, respiratory distress, metabolic acidosis (n=1) |
| Kappanayil *et al*, 2021^8^ | Cardiogenic hypotensive shock, respiratory distress, hepatomegaly, necrotic pressure ulcers over occiput and gluteal regions (n=1) |
| McCarty *et al,* 2021^10^ | Fever, PPHN, respiratory failure (n=1) |
| Schoenmakers *et al,* 2020^11^ | Hypotensive shock with multi-organ failure (raised creatinine, liver and cardiac enzymes), PPHN, bilateral intraventricular haemorrhage (grade 3 left sided; grade 2 right sided), coronary artery dilatation, hepatic and renal dysfunction (n=1) |
| Borkotoky *et al,* 2021^12^ | PPHN, respiratory distress, vasculitis rash, fever, necrotising enterocolitis (n=1) |
| Shaiba *et al,* 2021^13^ | PPHN, left ventricular systolic dysfunction, transaminitis (n=1) |
| Amonkar *et al,* 2021^14^ | Spontaneous aortic thrombosis resulting in right lower limb gangrene, irritability (n=1) |
| Diwakar *et al,* 2021^15^ | Fever, diarrhoea, rash over forehead and cheeks (n=1) |
| Costa *et al,* 2021^16^ | Multiple organ involvement, abnormal thickening of coronary artery walls (n=1) |
| Amulya *et al,* 2021^17^ | Fever, cough, rhonchi, respiratory distress, abnormal body movements, coronary artery dilatation (n=1) |
| Agrawal *et al,* 2021^18^ | Fever, lethargy, compensated shock, respiratory distress, intestinal dilatation, occipital ulcer (n=1) |
| Bakhle *et al,* 2022^19^ | Fever, lethargy, respiratory distress due to cavitary lung lesion (n=1) |
| Nitya *et al,* 2022^20^ | Poor activity, cold peripheries, and feeble peripheral pulses. In shock with erythematous rash over the eyelids, cheek, chest, and upper abdomen (n=1) |
| Sojisirikul *et al,* 2022^21^ | Abdominal distention and apnoea followed by respiratory distress, tachycardia, and tachypnoea (n=1) |
| Voddapelli *et al,* 2022^22^ | Fever, drowsiness, tachycardia, hypotension with cool peripheries, and tachypnoea with chest retractions. Developed abdominal distension with bilious vomiting and fever spikes (n=1) |
| Gupta *et al,* 2022^23^ | Refractory PPHN with persistent cardiac dysfunction and coagulopathy (n=1)  Unexplained severe cardiac dysfunction and hypertension on day 6 of life associated with aortic and intracardiac thrombosis (n=1) |
| Malek *et al*, 2022^24^ | Respiratory distress (tachypnea, chest indrawing, grunting with peripheral cyanosis) |
| Shinde *et al*, 2021^25^ | Respiratory distress; Poor perfusion, capillary refill time 5s, hypotension, tachycardia, cold extremities. Lethargic, seizures. |
| Aguilar-Caballero *et al*, 2023^26^ | Progressive respiratory deterioration |
| Arun *et al*, 2022^27^ | Fever; Poor feeding, lethargy, seizures, apnoea; Anemia, intramuscular hematoma, intracranial bleed |
| Ragireddy *et al,* 2023^28^ | Respiratory distress, Poor perfusion, Poor feeding, pulmonary arterial hypertension (n=1) |
| Rackauskaite *et al,* 2023^29^ | Myocarditis, supraventricular tachycardia, cardiogenic shock, hepatic injury, renal failure |
| Abdulaziz- Opiela *et al,* 2023^30^ | Respiratory distress; Embolism in arterial duct, left pulmonary artery, and pulmonary trunk, Pericardial effusion, Ascites, Hepatomegaly |
| Shanker *et al*, 2021^31^ | Fever, respiratory distress, shock, feeding difficulty, lethargy, seizure (1)  Feeding difficulty, lethargy (3) |
| More *et al,* 2022^32^ | Hypotensive shock, Respiratory distress, Encephalopathy (n=1)  Hypotensive shock, Respiratory distress, Refusal of feeds, Lethargy (n=1)  Hypotensive shock, Respiratory distress, Fever (n=1)  Apnoea, Refusal of feeds, Lethargy, Seizure (n=1)  Apnoea, Refusal of feeds, Lethargy, Fever (n=1)  Shock, Respiratory distress, Hypoglycaemia (n=1)  Respiratory distress, Refusal of feeds, Lethargy (n=1)  Respiratory distress, Refusal of feeds, Lethargy, Fever (n=1)  Refusal of feeds, Lethargy, Fever (n=2)  Refusal of feeds, Fever (n=1)  Respiratory distress (n=2)  Respiratory distress, Fever (n=1) |
| Pawar *et al*, 2021^33^ | Bradycardia with prolonged QTc and 2:1 AVB (n=4)  Respitory distress, bradycardia with prolonged QTc and 2:1 AVB (n=3)  Fever, hypotension, LV dysfunction (n=1)  Antenatal fetoplacental compromise, Shock, mild LV dysfunction, bilateral pleural effusions (n=1)  Antenatal fetoplacental compromise, bradycardia with prolonged QTc and 2:1 AVB (n=1)  Antenatal fetoplacental compromise, bradycardia with prolonged QTc and 2:1 AVB, feeding intolerance (n=1)  Antenatal fetoplacental compromise. Brownish gastric aspirates, frank melena, SVT, bilateral pleural and pericardial effusion (n=1)  Grunting, tachypnea, lethargy, feeding intolerance, intermittent bradycardia, hypotension (n=1)  Feed intolerance, decreased activity, brown gastric aspirates, rash, pedal edema, oral and skin lesions (n=1)  Feed intolerance, Cardiomegaly, cardiogenic shock, dilated coronaries, severe pulmonary arterial hypertension (n=1)  Seizures, shock, bradycardia, acute renal failure, mild LV dysfunction (n=1)  Fever, feeding intolerance, tachypnea, desaturation (n=1)  Antenatal pleural, pericardial effusions and ascites. Respiratory distress, shock; dilated hypertrophied RV with dysfunction, large thrombus at LPA origin (n=1)  Antenatal pleural, pericardial effusions, and ascites, pitting edema over chest wall, hepatomegaly, tachypnea, crepitations; dilated coronaries (n=1)  Mottling and poor peripheral pulsations, hypotension; intracardiac thrombus in right atrium (n=1) |
| Tambekar *et al*, 2022^34^ | Mild respiratory distress; Tachycardia, cold peripheries, capillary refill time> 3s, grade 2 murmur; Mild ascites with hepatic congestion (n=1)  Grade 2 murmur; Poor feeding (n=1)  Grade 4 murmur, tachycardia, poor perfusion; Poor feeding, absent suck; Lethargy, seizures, hypotonia, hyporeflexia. Maculopapular rash (1) |
| Saeedi *et al*, 2023^35^ | Diarrhoea; Dehydration; Fever; Multi-form macular rash on trunk and limbs (1)  Cough; Rashes on head/palms/soles; (n=1) |
| Balleda *et al*, 2022^36^ | Fever (n=18)  Respiratory distress (n=17)  Seizures (n=8)  Abdominal distension (n=3)  Skin rashes (n=2)  Coronary artery dilatation (n=8) |
| Chaudhuri *et al*, 2022^37^ | Severe respiratory distress, shock (n=1)  Thrombocytopenia Respiratory distress, coronary aneurysm, PPHN (n=1)  Respiratory distress; Dilated right heart with cardiomegaly, coronary aneurysm, PPHN, ventricular dysfunction (n=1)  PPHN, shock (n=2)  PPHN, coronary aneurysm (n=2)  Intermittent premature atrial ectopics, desaturation (n=1)  Cardiogenic shock, pneumothorax, ICH, hydrocephalous (n=1)  Cardiogenic shock (n=1)  Respiratory distress, oxygen dependency, large thrombus in left atrium, coronary aneurysm, mild PAH (n=1)  Cyanosis, severe PAH, moderate TR, right-to-left shunt across foramen ovale and bidirectional ductal shunt, cardiomegaly (n=1) |
| Hashiq *et al*, 2021^38^ | Hematemesis, Meconium Aspiration Syndrome, Respiratory distress (n=2)  Hematemesis, Respiratory distress (n=1)  Hematemesis, seizure (n=1) |
| Gamez- Gonzalez *et al,* 2022^39^ | Respiratory distress, Coronary artery dilatation, Pericardial effusion (n=1)  Respiratory distress, Coronary artery dilatation (n=1)  Respiratory distress, Pulmonary and tricuspid regurgitation, Finger desquamation (n=1) |
| Charki *et al,* 2022^40^ | Respiratory distress (n=56)  Cardiac dysfunction (n=46) PPHN (n=16) Fever (n=40) Seizures (n=15) Encephalopathy (n=7) Sepsis like (n=30) Hypoglycemia (n=4) Parotitis (n=4) |
