# Supplementary material for: Multisystem inflammatory syndrome in neonates (MIS-N): an updated systematic review
Source: Front Pediatr. 2024 Jul 4;12:1382133. doi: 10.3389/fped.2024.1382133 (PMC11256206; doi:10.3389/fped.2024.1382133)
Supplement: Supplementary file 4 [file Table4.docx]

**Supplementary Table 4.** Laboratory results of infants with MIS-N.

| **Study** | **Platelet abnormalities (x10^9^/L)** | **CRP (mg/dL)** | **Procal (ng/mL)** | **Ferritin (ng/mL)** | **LDH (U/L)** | **D-dimer (ng/mL**) | **IL-6 (pg/mL)** | **Transaminitis (IU/L)** | **Elevated cardiac enzymes** | **ECG findings** | **Echocardiography findings** |
| --- | --- | --- | --- | --- | --- | --- | --- | --- | --- | --- | --- |
| Divekar *et al*, 2021^7^ | Thrombocytopenia | Normal | NS | NS | NS | NS | 21.9 (0–15ng/mL) | NS | BNP >5,000 pg/mL  Troponin I = 0.189 ng/mL | Non-specific and non-diagnostic ST changes in the precordial leads | Pathological coronary artery dilatation with echo-bright walls (RCA 2.2 mm, LMCA 2 mm, LAD 1.3 mm), severe left ventricular systolic dysfunction (EF 35%), moderate mitral regurgitation, small pericardial effusion, elevated pulmonary artery pressures |
| Lima *et al,* 2020^8^ | No | Normal | NS | 358 (13-150) | 1161 (140-271) | 1551 (<500 µg/dL) | Yes | No | CPK = 695 U/L (<145)  CK = 41.6 ng/ml (<5)  Troponin = 0.314 ng/ml (<0.018) | Sinus rhythm | Pericardial effusion with dilatation of the vena cava discovered antenatally |
| Kappanayil *et al*, 2021^9^ | No | Normal | NS | 56 400 (12–327) | 6280 (0-451) | 2000 | NS | ALT 2240  AST 866 | NT-pro-BNP = 157000 pg/mL,  CKMB = 14·7 ng/mL    Troponin T = 1·23 ng/mL | Sinus rhythm | Severe biventricular dysfunction with LVEF of 10% and global hypokinesia, coronary arteries are normal in origin and luminal dimensions but appeared prominent and hyperechoic |
| McCarty *et al,* 2021^10^ | No initally | 6.78 (<1) | NS | NS | NS | NS | NS | NS | Nil | NS | Severe pulmonary hypertension |
| Schoenmakers *et al,* 2020^11^ | Thrombocytopenia | Normal | NS | 14272 | NS | Yes | NS | Yes | Yes | NS | Persistent pulmonary hypertension, flattened interventricular septum, mild to moderate tricuspid regurgitation, a small patent ductus arteriosus with predominantly right to left shunt and a significant enlarged left main coronary artery |
| Borkotoky *et al,* 2021^12^ | 85 (150-450) | 6.5 (<5) | NS | 1432 (12-327) | 702 (85-227) | >10000 (<50) | 43.49 (0-7) | NS | CK-MB = 41 (5–25 U/L)  HS Troponin-I = 171.2(<19 ng/L)  NT-pro BNP = 6125 (<62 pg/mL) | NS | Persistent pulmonary hypertension |
| Shaiba *et al,* 2021^13^ | No | No | 73.07 ng/ml | 384.4 | 2696 | NS | NS | NS | CK = 4,273 U/L  BNP = 3,433 pg/ml | NS | Moderately dilated left ventricle with poor systolic function, echogenic papillary muscles, wide patent ductus arteriosus with bidirectional shunt |
| Amonkar *et al,* 2021^14^ | No | 18.6 (<0.3) | 1.28 (<0.5) | 515 (21.8–274.6) | 448 (135-225) | 4110 (<500) | 20.29 (<7) | NS | NT-proBNP 12194 (<125 pg/mL) | NS | Acute thrombosis of lower abdominal aorta below renal arteries (80–90% occlusion) |
| Diwakar *et al,* 2021^15^ | Thrombocytopenia | 12.13 (0.08–1.12) | NS | 710.30 (25-300) | NS | 1620 (<500) | 1624 (0.5-6.4) | No | NS | Sinus rhythm | Normal |
| Costa *et al,* 2021^16^ | NS | NS | NS | NS | NS | NS | NS | NS | NS | NS | Severe coronary artery dilatation on echocardiography, however coronary artery internal diameters are normal on cardiac computed tomography angiography. Comparing the two imaging techniques, deduced to be an abnormal thickening of coronary arterial walls |
| Amulya *et al,* 2021^17^ | NS | Normal | NS | 902 (30-400) | 545 (135-255) | Normal | NS | NS | CKMB 94 (0–24 ng/mL)  Troponin T 0.177 (0–0.1 ng/mL)  CPK 376 (38–174 ng/mL) | NS | Coronary artery dilatation of LAD and RCA |
| Agrawal *et al,* 2021^18^ | No | Normal | 10.76 (<0.5) | Normal | 764 (10-25) | 1339 (0-500) | NS | ALT 42 (0-40)  AST 78 (0-40) | NT-Pro-BNP 4297 (<125 pg/mL) | Sinus rhythm | Normal |
| Bakhle *et al,* 2022^19^ | NS | 0.98 (<0.6) | 6.34 (0.1-4.2mg/L) | 412 (25-200) | 412 (290-775) | 4894 (<270) | NS | NS | NS | NS | NS |
| Nitya *et al,* 2022^20^ | NS | 4.7 (<0.6) | NS | 234 | 415 | 1690 | NS | NS | NT-pro-BNP 413 pg/mL | Bradycardia with prolonged corrected QT interval (QTc) (629 ms) | Normal |
| Sojisirikul *et al,* 2022^21^ | NS | Normal | Normal | 912 (25-200) | 989 (225-600) | 31191 (<500) | 14.69 (0-7) | NS | NS | Sinus tachycardia | Left ventricular ejection fraction of 33%, small peri-membranous ventricular septal defect, mild coarctation of the aorta, and moderate-to-severe tricuspid regurgitation |
| Voddapelli *et al,* 2022^22^ | NS | 61.6 | NS | 244 | 1086 | 4259 | 19.9 | NS | NT-Pro BNP: >35000 pg/mL | Sinus tachycardia | Dilated, hyperechogenic coronaries |
| Gupta *et al,* 2022^23^ | NS | 3.81 | NS | >1650 | 4402 | NS | NS | NS | Troponin T 0.22 ng/mL  NT-pro-BNP, > 35,000 pg/mL  CPK, 3985 U/L | NS | NS |
|  | NS | NA | NS | >1650 | 820 | >2000 | 25 | NS | Troponin T 0.09 ng/mL | NS | NS |
| Malek *et al*, 2022^24^ | 85 | Raised | Normal | 137 | NS | 2400 | 13.48 | NS | NS | NS | Moderate PPHN, PASP at 49 mmHg, moderate perimembranous VSDs, small PDA/ASD |
| Shinde *et al*, 2021^25^ | Normal | 1.72 (<0.6) | NS | 1115 (15-150) | 2477 (250-450) | >1200 (<252) | 72.21 (0-7) | NS | Trop T 1062 (<80pg/ml) | NSR | Mild LV dysfx with LVEF 40% |
| Aguilar-Caballero *et al*, 2023^26^ | NS | 3 (<1) | NS | NS | NS | NS | 36.2 (10x normal) | NS | NS | NS | Normal |
| Arun *et al*, 2022^27^ | NS | 1.2 (<1) | NS | 448 | 1071 | 973 | NS | NS | NS | NS | Prominent left coronaries with aneurysmal  dilatation and normal LVEF−77% |
| Ragireddy *et al,* 2023^28^ | NS | NS | NS | 579 | NS | 7080 | NS | NS | NS | NS | Severe pulmonary arterial hypertension (significant TR with pulmonary arterial peak systolic pressure of 53 mm of Hg) with LVEF of 44%. |
| Rackauskaite *et al,* 2023^29^ | NS | NS | NS | Raised | NS | Raised | Raised | NS | NS | SVT of 260-280bpm | Severely impaired left ventricle, LVEF 5%. |
| Abdulaziz- Opiela *et al,* 2023^30^ | 100 | 1.153 | NS | NS | NS | 7420 | NS | NS | NS | NS | Enlarged heart atria and a spherical structure with a diameter of 4.5 mm at the connection point between arterial duct and left pulmonary artery. Right ventricular systolic dysfunction. |
| Shanker *et al*, 2021^31^ | Thrombocytosis | Raised | NS | Normal | NS | Raised | NS | NS | NS | NS | NS |
|  | Thrombocytopenia | Raised | NS | Normal | NS | Raised | NS | NS | NS | NS | NS |
|  | 724 | 26.5 | NS | 1608 | NS | 16000 | NS | AST 262  ALT 92 | NS | NS | Biventricular dysfx - LVEF 28%; hyperechoic coronary arteries |
|  | Thrombocytosis | Raised | NS | Raised | NS | Raised | NS | NS | NS | NS | NS |
| More *et al,* 2022^32^ | Thrombocytopenia (n=2),  Thrombocytosis (n=3) | Raised (n=8), Normal (n=6) | Raised (n=6), Normal (n=8) | Raised (n=7), Normal (n=7) | Raised (n=4), Normal (n=10) | Raised (n=9), Normal (n=5) | Normal (n=1),  NS (n=13) | NS | Yes (n=7),  No (n=7) | NS | NS |
| Pawar *et al*, 2021^33^ | Thrombocytopenia (n=2),  NS (n=18) | Raised (n=14), Normal (n=6)  24  (9-62) [0-6] | Raised (n=6), Normal (n=14)  2.05 (1.3-5.1) [<0.5] | Raised (n=4), Normal (n=16) | Raised (n=8)  Normal (n=12)  1315 (793-6424) [290-775] | Raised (n=17)  Normal (n=3)  5932 (2820-12000) [<2700] | Raised (n=1),  NS (n=19) | NS | Yes (n=8),  No (n=12)  NT-Pro-BNP 24300 pg/mL (7361-30000) [<11,987 for 0-2 days, <5918 for 3-11 days] | Bradycardia with prolonged QTc and 2:1 atrioventricular block (n=9),  SVT (n=1) | Dilated coronaries (n=2),  Intracardiac thrombus (n=2),  Cardiac dysfunction (n=3),  Pleural/pericardial effusions (n=2) |
| Tambekar *et al*, 2022^34^ | 47 | Raised | NS | 245 (15-200) | 369 (10-25) | 2100 (<500) | NS | NS | Troponin T 0.5 ng/ml (0.01-0.06 ng/ml) | NS | Coronary aneurysms; RCA=1.9 mm (+4.3); LMCA=1.8 mm (+3.2), trace pericardial collection with 45-50% LVEF |
|  | 47 | Raised | NS | 125 (15-200) | 2373 (10-25) | 1160 (<500) | NS | NS | Troponin T (0.09 ng/ml) | NSR | PDA (2 mm), dilated coronaries RCA=1.6 mm (+2.5); LMCA=1.4 mm (+1.4), normal LVEF |
|  | Thrombocytopenia | Raised | NS | 340 (15-200) | 1118 (10-25) | 7940 (<500) | NS | NS | NA | NS | Large peri membranous VSD, ASD (4 mm), dilated RA and RV |
| Saeedi *et al*, 2023^35^ | Normal | 93 | NS | NS | NS | 1215 | NS | Normal | NS | NS | NS |
|  | Normal | 2 | NS | NS | NS | 435 | NS | Normal | NS | NS | NS |
| Balleda *et al*, 2022^36^ | NS | Raised (n=8) Normal (n=10)  1.913 (0.3-1.139) [<0.6] | Raised (n=5) Normal (n=13)  15.04 (0.1-85) [<1] | Raised (n=6) Normal (n=12)  300.81 (68-607.32) [<200] | Raised (n=18)  998.44 (607-1855) [<280] | Raised (n=18)  4740.4 (670 – 10000) [<500] | NS | NS | NS | NS | Coronary dilatations (n=10)  Pulmonary HTN (n=4) |
| Chaudhuri *et al*, 2022^37^ | Thrombocytopenia (n=8) | Raised (n=12)  4.99 (1.39-11) [<1] | NS | NS | Raised (n=4)  Normal (n=8)  2465 (436-5864) [160-1500] | Raised (n=10)  8.405 (1.89-37.76) [<0.5] | NS | Yes (n=8)  No (n=4) | Yes (n=9)  No (n=3) | Sinus tachycardia (n=11)  Atrial bigeminy (n=1) | Cardiomegaly (n=12)  Severe right heart enlargement, proportionate to the severity of PPHN (n=9)  Reduced LVEF<55% (n=5)  Pulmonary arterial HTN (n=10)  Coronary artery dilatation (n=7)  Giant left atrial thrombus (n=1)  Mean LVEF 49.5% (range 10%–60%). Cardiac dysfunction |
| Hashiq *et al*, 2021^38^ | NS | 30 | NS | 973 | 2353 | 7710 | NS | NS | NS | NS | NS |
|  | NS | 48 | NS | 983 | 3450 | 8200 | NS | NS | NS | NS | NS |
|  | NS | 35 | NS | 873 | 4340 | 6780 | NS | NS | NS | NS | NS |
|  | NS | 42 | NS | 1042 | 2950 | 7402 | NS | NS | NS | NS | NS |
| Gamez- Gonzalez *et al,* 2022^39^ | 457 | Normal | NS | 1740 | NS | NS | NS | NS | CK-MB 26.3 U/L | NS | LVEF 61% and minimal pericardial effusion with coronary artery dilation |
|  | Normal | Normal | NS | 3650 | NS | 1983 | NS | NS | CK- MB 22 U/L | NS | Normal biventricular systolic function but identified coronary artery dilation, and hypertrophic myocardiopathy |
|  | 30 | 0.256 | NS | NS | NS | NS | NS | Normal | Troponin 84.3ng/mL  Pro-BNP 12,700 pg/mL | NS | Tricuspid and pulmonary regurgitation with normal ventricular function. |
| Charki *et al,* 2022^40^ | Thrombocytosis (n=12), Thrombocytopenia (n=18) | Raised (n=45), Normal (n=53) | NS | Raised (n=45), Normal (n=53) | Raised (n=35) Normal (n=63) | Raised (n=40) Normal (n=58) | NS | NS | Raised (n=50), Normal (n=48) | NS | LV dysfunction (n=46), Dilated coronaries (n=8), PPHN (n=16), Pericardial effusion (n=4) |

CK: Creatine Kinase; CKMB: Creatine kinase-myoglobin binding; CRP: C-reactive protein; ECG: Electrocardiogram; EF: Ejection Fraction; LAD: Left Anterior Descending artery; LMCA: Left main coronary artery; LVEF: Left Ventricular Ejection Fraction; MIS-N: Multisystem Inflammatory Syndrome in Neonates; NS: Not Stated; NT Pro-BNP: N-terminal pro b-type natriuretic peptide; Procal: Procalcitonin; RCA: Right Coronary Artery; ST: ST segment in ECG.
