# Supplementary material for: Multisystem inflammatory syndrome in neonates (MIS-N): an updated systematic review
Source: Front Pediatr. 2024 Jul 4;12:1382133. doi: 10.3389/fped.2024.1382133 (PMC11256206; doi:10.3389/fped.2024.1382133)
Supplement: Supplementary file 5 [file Table5.docx]

**Supplementary Table 5.** Therapeutic management and outcomes of infants with MIS-N.

| **Study** | **IVIG (dose)** | **Corticosteroid** | **LMWH** | **Inotropic support** | **Respiratory support** | **Miscellaneous treatment** | **No. of days hospitalised** | **Time taken for improvement in inflammatory markers (days)** | **Days on inotropic support; Cardiac improvement** | **No. of days intubated** | **Outcome** |
| --- | --- | --- | --- | --- | --- | --- | --- | --- | --- | --- | --- |
| Divekar *et al*, 2021^7^ | Yes (NS) | Hydrocortisone | No | Dopamine (3mcg/kg/min) | Surfactant, High frequency ventilation | Antibiotics, Fresh frozen plasma, Packed cell transfusion, Cryoprecipitate | 60 | NS | Ventricular function improved 16H post-IVIG, Coronary dilatation resolved 8 days post-IVIG | Weaned from high frequency ventilation to conventional ventilation 72H post-IVIG and to NIV 8 days post-IVIG | Survived; standard course of preterm infant apart from severe retinopathy |
| Lima *et al,* 2020^8^ | No | No | No | Yes | Mechanical ventilation | Pericardiocentesis and amine infusion, Antibiotics | 22 | 5 | 5; Repeat echo showed resolution of pericardial effusion | 7 | Survived |
| Kappanayil *et al*, 2021^9^ | Yes (2 g/kg once) | Methylprednisolone (5 mg/kg once daily for 3 days) | 10 U/kg/h and titrated | Epinephrine  (0.04 μg/kg/min), Milrinone (0·5 μg/kg/min) | Mechanical ventilation | Diuretics, Antibiotics, Calcium gluconate, Vitamins C and D, Zinc | 29 | 2 | 13; echo at discharge showed improved LFEV 58%; coronaries less hyperechoic | 11 | Survived |
| McCarty *et al,* 2021^10^ | No | No | No | No | Mechanical ventilation, 2 doses surfactant | Antibiotics, Inhaled nitric oxide for PPHN | 22 | 8 | PPHN resolved on echo on D4OL | 5 | Survive; no follow-up required aside from standard prematurity care |
| Schoenmakers *et al,* 2020^11^ | Yes (2 g/kg) | Hydrocortisone | No | Yes | Mechanical ventilation | Inhaled nitric oxide, Aspirin, Antibiotics | NS | 6 | 6; Echo on D14OL showed only mild LMCA dilatation | 6 | Survived |
| Borkotoky *et al,* 2021^12^ | No | Dexamethasone | No | Dopamine | Mechanical ventilation | Antibiotics, Diuretics, Sildenafil | 34 | 24 | NS; echo at discharge showed resolution of PPHN | 16 | Survived; well at follow-up at age 2 months |
| Shaiba *et al,* 2021^13^ | Yes (2 doses of 1g/kg) | Hydrocortisone (0.5 mg/kg q12H x7/7 then q24H x3/7) | Nil | Dobutamine | HFOV | Antibiotics, inhaled nitric oxide for PPHN | NS | 5 | 3; echo on D4OL showed improved LVEF 68% and closed ductus arteriosus | 6 | Survived |
| Amonkar *et al,* 2021^14^ | No | Dexamethasone 0.15 mg/kg/day | Unfractionated heparin (bolus 80 U/kg, then 28 U/kg/h) | No | Ventilatory support (not specified) | Surgical embolectomy, followed by right lower limb amputation. Antibiotics. Aspirin. rTPA | 28 | 21 | NA | NS | Survived, right below knee amputation but no other sequalae |
| Diwakar *et al,* 2021^15^ | Yes (2g/kg) | No | No | No | No | No | 6 | 5 | NA | NA | Survived; well at 5-week follow up |
| Costa *et al,* 2021^16^ | NS | NS | NS | NS | NS | NS | NS | NS | NS | NS | Survived |
| Amulya *et al,* 2021^17^ | Yes (NS) | Methylprednisolone | No | No | NIV | Antibiotics, aspirin, anti-convulsant | NS | NS | Echo on first day of life showed resolution of coronary artery dilatation | NA | Survived |
| Agrawal *et al,* 2021^18^ | Yes (2g/kg q12H) | Methylprednisolone 1mg/kg q12H | SC enoxaparin (1mg/kg BD) | Dopamine 10mcg/kg/min | Mechanical ventilation | Antibiotics, Aspirin | 16 | NS | 2 | 9 | Survived; well at 45-day follow-up |
| Bakhle *et al,* 2022^19^ | Yes (1g/kg/day) | No | No | No | No | Antibiotics | NS | NS | NA | NA | Survived, well at 3-month follow-up |
| Nitya *et al,* 2022^20^ | No | IV 5 mg/kg/day | No | No | No | NS | 6 weeks | 7 | NS | NS | Survived |
| Sojisirikul *et al,* 2022^21^ | Yes (2g/kg, 2 divided doses) | IV 2mg/kg/day for 5 days | No | Yes | Yes | NS | 86 | 1 | 12 | 12 | Survived |
| Voddapelli *et al,* 2022^22^ | Yes (1 g/kg/day for 2 days) | No | No | Yes | Yes | NS | 10 | 2 | 5 | 5 | Survived |
| Gupta *et al,* 2022^23^ | Yes (n=2); dose NS | No | No | Yes (n=2) | Yes (n=1) | NS | NS | NS | NS | NS | Survived (n=1),  Deceased (n=1) |
| Malek *et al*, 2022^24^ | Yes, 5 days | No (thrombocytopenia) | NA | Pulmonary vasodilator, Sildenafil | CPAP | Antibiotics, FFP | 24 | 15 | NA | NA | Survived |
| Shinde *et al*, 2021^25^ | 2g/kg | 2mg/kg/day x14/7 | No | inotropic support | CPAP, intubated and ventilated | Phenobarbitone, Keppra, fluid resus, FFP, PCT | 3 | 48H | 72H | NA | Survived |
| Aguilar-Caballero *et al*, 2023^26^ | NA | Dexamethasone x 2 courses | NA | NA | CPAP, intubated, ventilated | Remdesivir (5 mg/kg loading dose, followed by 2.5 mg/kg/dose daily x4/7) Antibiotics for VAP | 150 | NA | NA | NA | Deceased; Progressive respiratory deterioration 2' to severe bronchopneumonia |
| Arun *et al*, 2022^27^ | 1 g/kg for 2days | 1mg/kg every 12h for 6 days and tapered  over 4 days. | NA | NA | Intubated | Factor VIII 125IU twice a day  (targeting 100%  factor levels),  anticonvulsants,  and antibiotics | NA | NA | NA | Day 6 | Survived, well at 2-months follow up |
| Ragireddy *et al,* 2023^28^ | 2g/kg for 48 hrs | 2 mg/kg/day  for 5 days  and tapered  in 10 days. | Injection enoxaparin 1mg/kg/day for 14 days | Nil | Nil | Nil | Day 22 | NA | NA | NA | Survived |
| Rackauskaite *et al,* 2023^29^ | 2g/kg for 48 hrs | 2 mg/kg | Nil | Nil | Nil | Aspirin | Day 5 (to  normal ward) | NA | NA | NA | Survived |
| Abdulaziz- Opiela *et al,* 2023^30^ | Nil | Nil | 2x 4.5mg LMWH | NA | Intubation | Antithrombin III was supplemented | 12th day of life | NA | NA | NA | Survived |
| Shanker *et al*, 2021^31^ | NA | NA | NA | NA | NA | NA | NA | NA | NA | NA | NA |
|  | NA | NA | NA | NA | NA | NA | NA | NA | NA | NA | NA |
|  | NA | 5mg/kg x3 days | 10u/kg | Epinephrine 0.2mcg/min | Mechanical ventilation | NA | NA | 48H | NA | 72H | Survived |
|  | NA | NA | NA | NA | NA | NA | NA | NA | NA | NA | NA |
| More *et al,* 2022^32^ | Yes (n=5) [dose NS], No (n=9) | Yes [Methylprednisolone, Dexamethasone] (n=11),  No (n=3) | Yes (n=1), No (n=13) | Yes [Dobutamine, Epinephrine, Norepinephrine, Milrinone, Vasopressin (n=6),  No (n=8) | Yes [Ventilation, HFNC, Surfactant] (n=5);  No (n=9) | Antibiotics (n=14),  Aspirin (n=2) | NS | NS | NS | NS | Survived (n=12), Deceased (n=2) |
| Pawar *et al*, 2021^33^ | Yes (n=20) [1-2g/kg] | Methylprednisolone 2 mg/kg/day (n=20) | 1.5 mg/kg, twice a day (n=14),  No (n=6) | Yes [Milrinone, Adrenaline, Dobutamine, Dopamine] (n=12),  No (n=8) | Yes [Mechanical ventilation, CPAP, Surfactant] (n=13),  No (n=7) | Aspirin, Alteplase, Betablockers, Lasix, Beta blockers | NS | NS | NS | NS | Survived (n=18), Deceased [NEC, Multi-organ failure] (n=2) |
| Tambekar *et al*, 2022^34^ | 2g/kg | 2mg/kg/dose | No | Yes | Nil | FFP, platelet infusion | 15 | Not stated | Not stated | NA | Survived |
|  | 2g/kg | 2mg/kg/dose | 1mg/kg | Nil | Nil | Aspirin | 13 | Not stated | Nil | Nil | Survived |
|  | 2g/kg | 2mg/kg/dose | No | Dobutamine 10 μg/kg/min, then dopamine 10 mcg/kg/min, then noradrenaline 0.2 mcg/kg/min | Intubated and ventilated | Keppra, glucose infusion, Lasix, antibiotics | Deceased | NA | NA | NA | Deceased; Multiorgan involvement and shock, profuse pulmonary haemorrhage |
| Saeedi *et al*, 2023^35^ | NA | IV MP 30mg/kg stat > 1mg/kg PO Prednisolone | NA | NA | NA | Antibiotics, fluids | Not stated | NA | NA | NA | Survived, well at 3m follow up |
|  | NA | Single dose of hydrocortisone | NA | NA | NA | NA | 25 (no ICU stay) | Cough and rash resolved in 1 day with hydrocortisone | NA | NA | Survived, well at 3m follow up |
| Balleda *et al*, 2022^36^ | 2g/kg (n=18) | IV Dexamethasone 0.15mg/kg (n=18) | 1 unit/kg (n=9) | NA | Ventilator/ CPAP/ HFNC/ nasal oxygen (n=17) | Aspirin 3mg/kg (n=15) | Mean 10.17 (Range 4 - 21) | NA | NA | NA | Survived (n=17)  Deceased (n=1) |
| Chaudhuri *et al*, 2022^37^ | Yes (n=10) | Yes (n=10) | 1 mg/kg twice daily (some) | NA | Intubation | Antibiotics | NA | 3 days post  IVIG/steroid | NA | NA | Survived (n=11)  Deceased (n=1)  - extreme preterm, died from NEC on  D20OL |
| Hashiq *et al*, 2021^38^ | Yes (n=4) | Nil | Yes (n=4) | NA | Intubation (n=4) | Antibiotics (n=4) | NA | NA | NA | NA | Survived, labs normalised (n=4) |
| Gamez- Gonzalez *et al,* 2022^39^ | 2g/kg | Methylprednisolone 1mg/kg/day | Enoxaparin | NA | Intubation | Aspirin | 15 | NA | NA | NA | Survived |
|  | 2g/kg | Methylprednisolone 1mg/kg/day | Enoxaparin | NA | Intubation | Aspirin | NA | NA | NA | NA | Survived but  had neurologic complications and severe prematurity retinopathy |
|  | 2g/kg | Methylprednisolone 3mg/kg/day | Nil | Dobutamine 5mcg/kg/min | Nil | Aspirin | NA | NA | NA | NA | Survived |
| Charki *et al,* 2022^40^ | Yes (n=26) [dose NS], No (n=72) | Yes (n=26) | No | Inotropes (n=28) Sildenafil (n=12) | NIV (n=27) Ventilation (n=16) Nasal oxygen (n=14) | NS | NS | Discharged (n=95) Deceased (n=3) | In severely affected neonates with cardiac dysfunction, most neonates showed improvement in clinical and biochemical parameters after both steroids and IVIG administration. | NS | Discharged (n=95) Deceased (n=3) |

CPAP: Continuous Positive Airway Pressure; FFP: Fresh frozen plasma; HFNC: High-flow nasal cannula; HFV: High-frequency ventilation; IVIG: Intravenous immune globulin; LMCA: Left Main Coronary Artery; LMWH: Low Molecular Weight Heparin; LVEF: Left Ventricular Ejection Fraction; MIS-N: Multisystem Inflammatory Syndrome in Neonates; NEC: Necrotizing enterocolitis; NIV: Non-invasive ventilation; NS: Not Stated; PPHN: Persistent Pulmonary Hypertension of the Newborn; SC: Subcutaneous; VAP: Ventilator associated pneumonia
